# Estimated Roles of the Carrier and the Bacterial Strain When Methicillin-Resistant Staphylococcus aureus Decolonization Fails: a Case-Control Study

**DOI:** 10.1128/spectrum.01296-22

**Published:** 2022-08-24

**Authors:** Mona Katrine Alberthe Holm, Kristina Melbardis Jørgensen, Kristian Bagge, Peder Worning, Michael Pedersen, Henrik Westh, Jonathan M. Monk, Mette Damkjær Bartels

**Affiliations:** a Department of Clinical Microbiology, Copenhagen University Hospital—Amager and Hvidovre, Copenhagen, Denmark; b Department of Clinical Medicine, University of Copenhagen, Copenhagen, Denmark; c Systems Biology Research Group, Department of Bioengineering, University of California, San Diego, San Diego, California, USA; University of Calgary

**Keywords:** methicillin-resistant *Staphylococcus aureus*, chronic carrier, treatment failure, MRSA decolonization, MRSA eradication

## Abstract

Methicillin-resistant Staphylococcus aureus (MRSA) is a common bacterial pathogen that frequently colonizes healthy individuals, with potential to cause invasive infection. In Denmark, to keep the prevalence low, MRSA carriers are recommended to undergo decolonization treatments, but achieving decolonization is challenging. Knowledge about the factors contributing to decolonization is scarce. We aimed to identify bacterial genome and clinical factors influencing MRSA decolonization. We identified all new MRSA patients above 2 years of age within the Hvidovre catchment area, Copenhagen, Denmark, in 2017 and 2018. Carriers were defined as chronic carriers (cases) if they were MRSA positive after two or more treatments and as nonchronic carriers (controls) if they were MRSA free after the first or second treatment. Using whole-genome sequencing (WGS), we constructed a pangenome of bacterial strains. With the incorporation of bacterial genome and clinical patient data, machine learning and multivariate analyses were performed to determine the factors associated with decolonization. A total of 477 MRSA carriers were included. An age of ≥13 years was significantly associated with nonchronic carriage. We identified 278 bacterial genetic features that were statistically significantly associated with chronic carriage (*P* < 0.05 by Fisher’s exact test). Chronic MRSA carriage was predicted with 68% accuracy using a combination of bacterial genome data and patient clinical data. Decolonization success is multifactorial. Apart from the 68% predicted accuracy found in this study, we estimate that the remaining 32% is a result of host factors and microbiome composition.

**IMPORTANCE** Carriage of methicillin-resistant Staphylococcus aureus (MRSA) and other multiresistant bacteria is a prerequisite for infection and transmission. Successful decolonization treatment removes these risks. We aimed to identify bacterial genome and host clinical factors that influence MRSA decolonization to estimate the roles of the carrier and the bacterial strain, respectively, when decolonization fails. The long-term goal, beyond this study, is to optimize decolonization success, minimize MRSA transmission, and, ultimately, improve the quality of life of MRSA carriers.

## INTRODUCTION

Staphylococcus aureus is a human commensal, permanently colonizing approximately 20 to 30% of the adult population, while part of the population is susceptible to short-term colonization (1). A number of bacterial colonization factors have been identified to be involved in S. aureus nasal colonization (2, 3). S. aureus carriers are at risk for a broad variety of subsequent infections with their own commensal strain. Genotyping has revealed that 80% of cases of bacteremia in S. aureus carriers were endogenous (4). It has been shown that carriers have significantly higher survival rates than noncarriers, ascribed to improved immune responses to the carriers’ commensal strain (5).

Methicillin-resistant S. aureus (MRSA) was first identified 60 years ago (6) and has developed from one initial clone into a multitude of clones geographically dispersed (7). MRSA can cause invasive infections, similar to methicillin-sensitive S. aureus (MSSA), but healthy asymptomatic individuals are often unaware of their MRSA carriage, with the possibility of transmitting MRSA (8). To prevent spread and maintain a low prevalence in Denmark, the Danish Health Authority (DHA) recommends that MRSA carriers and all household contacts go through decolonization treatment (9).

Decolonization is a challenge. We have previously found that multiple decolonization treatments are often necessary, and a treatment success rate of 61.6% was found after up to four treatments (10). In Denmark, two negative sets of control swabs from the nose, throat, and perineum, 1 and 6 months after decolonization treatment, are required for an individual to be declared MRSA free. Although most carriers are asymptomatic, it has been found that 33% experience MRSA-associated stigma, as they feel that they are treated differently because of their MRSA status, contributing to poor mental health (11).

The purpose of this study was to analyze clinical data and MRSA genomic data to uncover factors that could be early predictors of postdecolonization chronic MRSA carriage. Knowledge of predictors of chronic carriage could improve the information for MRSA carriers about the expected duration of carriage and lead to the optimization of individualized decolonization treatments and, ultimately, an improved quality of life.

## RESULTS

**FIG 1:**
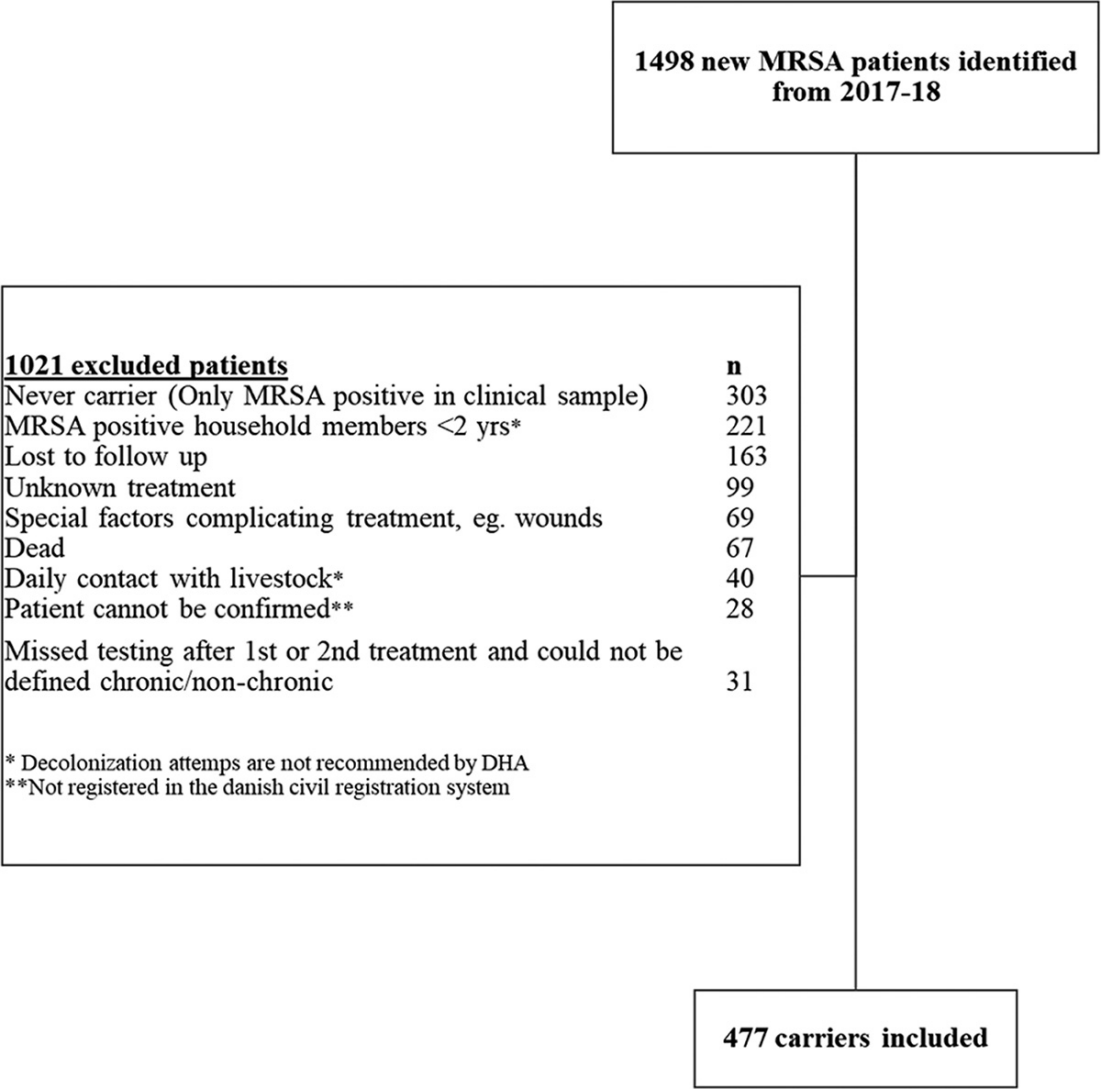
Flow diagram of all 1,498 identified MRSA patients from 2017 to 2019. A total of 477 carriers were eligible to be included in the study; the remaining patients were excluded.

A total of 1,498 new MRSA patients were identified in 2017 and 2018 from the Hvidovre Hospital catchment area. We included 477 carriers in the study, while 1,021 were excluded (Fig. 1). A total of 312 (65%) were nonchronic carriers, and 165 (35%) were chronic carriers.

### Clinical data.

**FIG 2:**
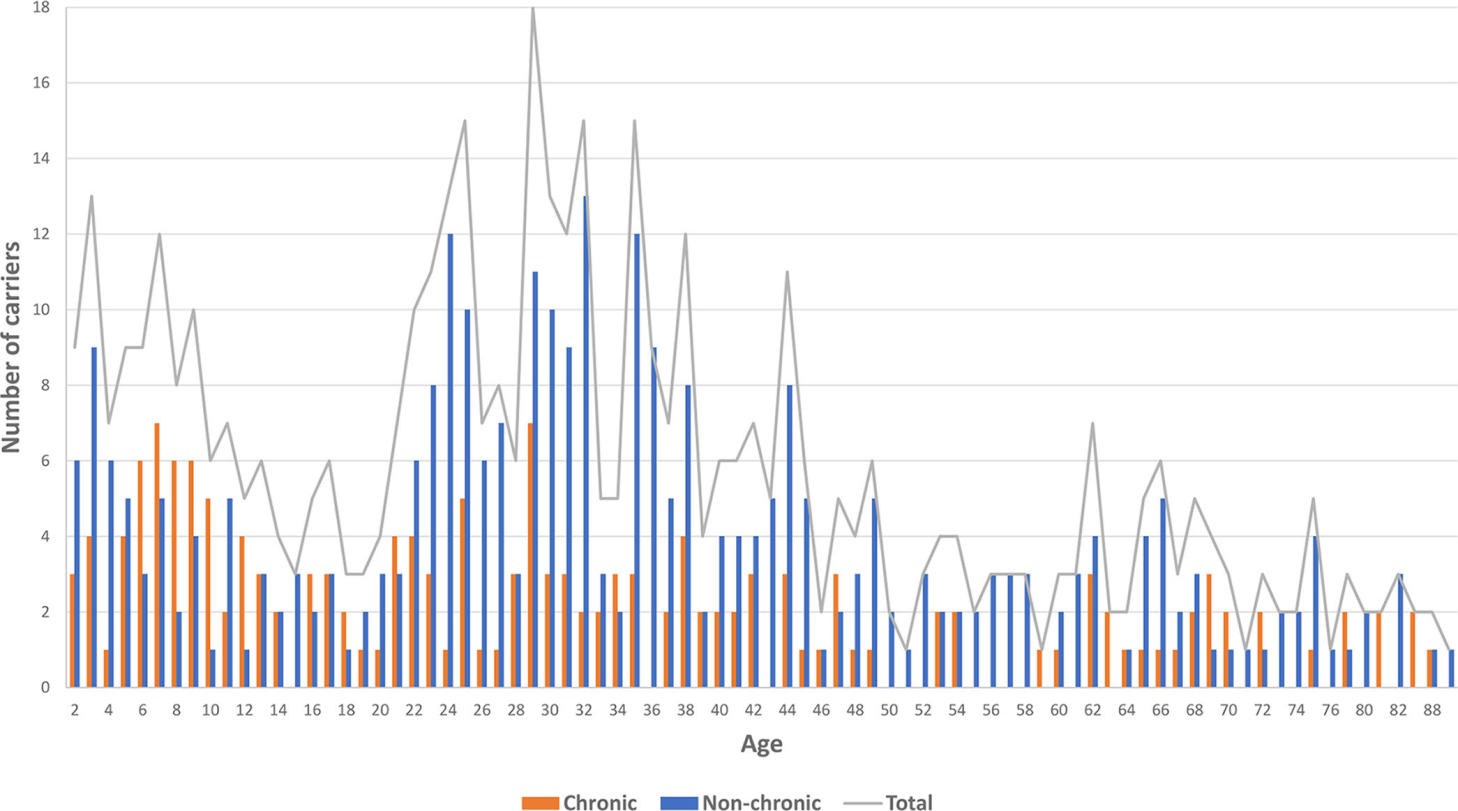
Age distribution of the included carriers. The mean age at the first positive sample was 31 years (range, 2 to 98 years). The two youngest age groups were associated with chronic carriage (*P* < 0.01).

See Table 1 for an overview of the two groups. The group of carriers that were positive only in the nose, 84/477 (18%), had the lowest rate of chronic carriage, with only 3/84 (6%) becoming chronic carriers (Table 1). This was followed by perineum-positive-only carriers, 32/477 (7%), with 3/32 (9%) becoming chronic carriers. A total of 93/477 (19%) were only throat positive, of whom 35/93 (38%) became chronic carriers. Carriers who were MRSA positive in the throat and any other screening location(s), 229/477 (48%), were most likely to become chronic carriers, with 115/229 (50%) becoming chronic carriers. Among chronic carriers, 155/165 (94%) were throat positive, while only 176/312 (56%) of the nonchronic carriers were throat positive. Among all carriers, 119/477 (25%) were less than 18 years of age, while 358/477 (75%) were 18 years of age or older (Fig. 2). The median age at the first positive sample was 31 years (range, 2 to 98 years).

**TABLE 1:** Overview of the two carrier groups

Parameter	Chronic carriers (*n* = 165)		Nonchronic carriers (*n* = 312)	
	No. of subjects	% of subjects	No. of subjects	% of subjects
Age group				
1 (2–12 yrs)	48	29	47	15
2 (13–22 yrs)	23	14	28	9
3 (23–45 yrs)	56	34	160	51
4 (>46 yrs)	38	23	77	25

MRSA-positive site before 1st treatment				
Nose	96	58	201	64
Throat	109	66	142	46

Clindamycin administration				
1st treatment	8	5	15	5
2nd treatment	27	16	42	13

Male sex	77	47	120	38

Household size (no. of members)				
1	17	10	37	12
2	38	23	95	30
3	26	16	48	15
4	42	25	73	23
>4	38	23	50	16

MRSA-positive site				
Only nose	3	2	81	26
Only throat	35	21	58	19
Only perineum	3	2	29	9
Throat + more	115	70	114	37

During the first decolonization treatment, 23/477 (5%) carriers, all above the age of 18 years, had clindamycin added to the standard decolonization treatment (Table 1). During the second treatment, 69/272 (25%) had clindamycin added to the standard treatment; 61/69 (89%) were above 18 years of age, and 8/69 (12%) were below 18 years of age, of whom 2 belonged to the youngest age group.

The cumulative decolonization rates after treatments were 43% after one treatment, 65% after two treatments, 71% after three treatments, and 75% after four treatments. The rate of decolonization including those who were spontaneously MRSA free at the end of the study period was 81%.

Multivariate logistic regression analysis identified age groups of 13 years of age and older as being significantly associated with nonchronic carriage (see Fig. 2 for the distribution of ages and carrier status) (age of 2 to 12 years, *P* = 0.22 [95% confidence interval {CI}, 0.09 to 1.75]; age of 13 to 22 years, *P* = 0.03 [95% CI, 0.12 to 0.92]; age of 23 to 45 years, *P* < 0.001 [95% CI, 0.09 to 0.43]; age of 46 years and above, *P* = 0.01 [95% CI, 0.11 to 0.75]).

There were no statistically significant differences in decolonization rates among carriers who were MRSA positive in the nose (*P* = 0.82 [CI, 0.66 to 1.69]) or throat (*P* = 0.23 [CI, 0.83 to 2.07]) or among carriers treated with clindamycin during the first (*P* = 0.56 [CI, 1.45 to 5.80]) or second (*P* = 0.69 [CI, 0.23 to 2.67]) treatment. Gender was not implicated in chronic carriage (*P* = 0.66 [CI, 0.43 to 1.70]), and household size did not significantly influence chronic carriage (*P* = 0.06 [CI, 1.00 to 1.35]), although we did see a trend toward increasing numbers of chronic carriers with increasing household sizes. Due to our inability to determine household members, nine nonchronic carriers and four chronic carriers were removed from the multivariate analysis as they were either homeless, jail inmates, or nursing home residents.

### Genomic data.

Whole-genome sequencing (WGS) was conducted on the first strain from each of the 477 carriers. We found 56 different sequence types (STs), of which the most prevalent were ST6, ST22, ST30, ST5, ST1, ST8, ST59, and ST772, with none being statistically associated with chronic carriage. A minimum-spanning tree (MST) based on core-genome multilocus sequence typing (cgMLST) of all isolates (see Fig. S1 in the supplemental material) showed no correlation with chronic carriage, as isolates from chronic and nonchronic carriers are scattered throughout the branches.

**FIG 3:**
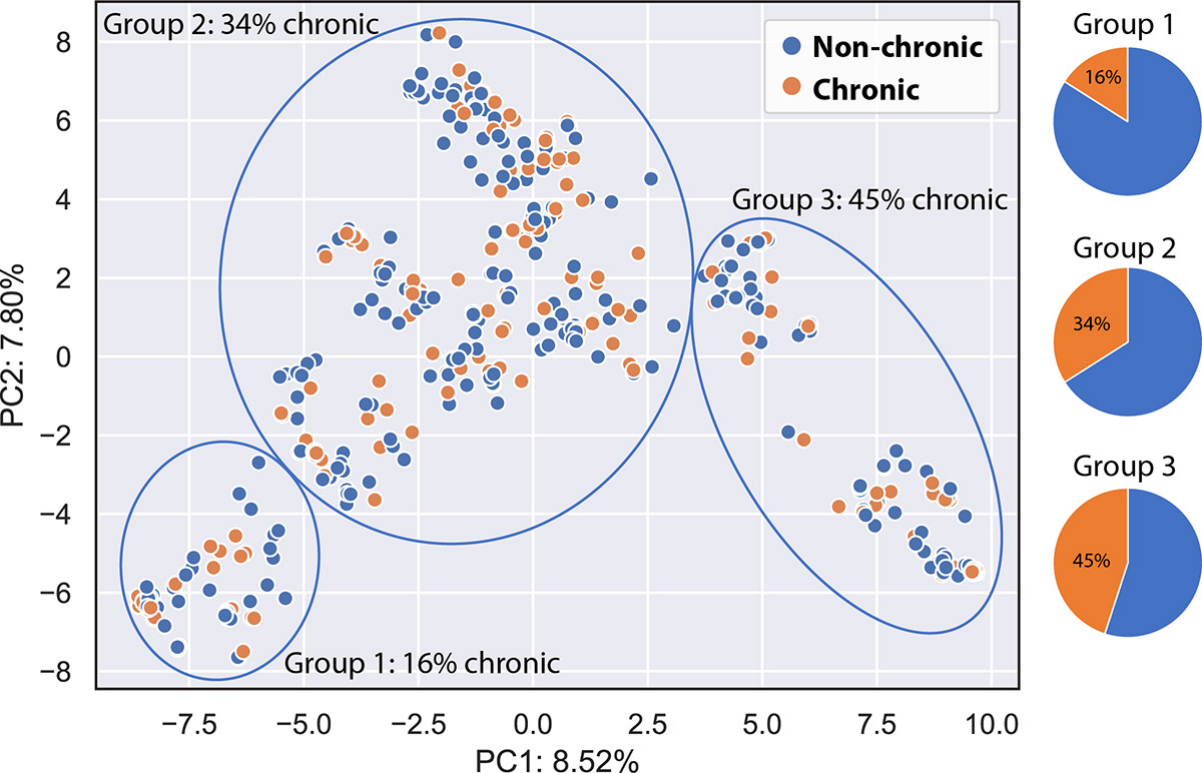
Multiple-correspondence analysis (MCA) plot of all 477 MRSA strains. Accessory genes are colored by MRSA status (orange, chronic; blue, nonchronic). Agglomerative clustering into three groups, with principal components PC1 and PC2 displaying isolates in relation to each other based on genetic resemblance, indicates correlations between bacterial genomic data and chronic carriage.

A pangenome of the strains was constructed to evaluate shared and unique genes among the strains. The total size of the pangenome was 5,295 genes, with a core-genome (genes shared by all strains) size of 1,671 genes, while the accessory genome (genes found in some strains) consisted of 3,624 genes. To determine whether chronic carriage may be associated with strain genetic factors, a multiple-correspondence analysis (MCA) was performed on a binary representation of the pangenome (Fig. 3). Agglomerative clustering was used to group strains showing an increase in chronic carriers along the *x* axis. Based on these results, we performed a pangenome-wide association analysis. A total of 278 genetic features were found to be associated with chronic or nonchronic carriage (Table S1) (*P* < 0.05 by Fisher’s exact test). The top 20 features are presented in Table 2.

**TABLE 2:** Top 10 chronic, followed by top 10 nonchronic, statistically significant gene products (*P* < 0.05) and their possible function, ranked by statistical association^*a*^

Gene product (NCBI protein accession no.)	Gene name	Possible function(s) (reference[s])	% chronic present	% nonchronic present	Carriage association
DNA invertase Hin (WP_000690626.1)		Initiation of DNA cleavage and recombination (20)	86	73	Chronic
Thiolase-like protein (WP_000894158.1)		Conversion of 2 U of acetyl-CoA to acetoacetyl CoA in the mevalonate pathway and isoprenoid biosynthesis regulation (21)	99	93	Chronic
Membrane protein (WP_000838599.1)		Serves multiple different functions, including transportation in and out of cells, binding of molecules, surface receptors, and involvement in intra- and extracellular signaling (36)	21	11	Chronic
HTH-type transcriptional regulator CysL (WP_000377738.1)		Transcriptional activator of the *cysJI* operon, involved in sulfur assimilation; negative regulation of its own transcription^*b*^	21	11	Chronic
Putative antitoxin YezG (WP_000142122.1)		YeeF/YezG is a toxin-immunity protein pair; YezG neutralizes the YeeF-CT DNase; YezG protects inhibitor cells from autoinhibition and protects cells from DNA degradation (37)	87	75	Chronic
CAAX amino protease (WP_001080636.1)		Metalloendopeptidase activity^*c*^	42	29	Chronic
Hydroxyacylglutathione hydrolase (WP_000184398.1)		Detoxification enzyme belonging to the broader metallo-β-lactamase family; acts to remove the toxic metabolite methylglyoxal and related compounds^*d*^	55	42	Chronic
Putative hydroxymethyl pyrimidine (HMP)/thiamine import ATP-binding protein YkoD (WP_000138663.1)		Part of an ABC transporter complex, which possibly transports hydroxymethylpyrimidine, thiamine, or similar products and may be responsible for energy coupling^*e*^	85	74	Chronic
*N*-Acetyltransferase (WP_000610979.1)		Generic *N*-acetyltransferase that needs deeper functional characterization^*f*^; other acetyltransferase genes (e.g., *speG*) have been associated with polyamine resistance (38)	85	74	Chronic
Putative HMP/thiamine permease protein YkoC (WP_001077829.1)		Part of an ABC transporter complex, which possibly transports hydroxymethylpyrimidine, thiamine, or similar products, and is likely responsible for substrate translocation across the membrane^*g*^	85	74	Chronic

Chemotaxis inhibitory protein (WP_000727649.1)	*chp*	Able to escape the innate immune system and contribute to colonization or early stage of infection; inhibition of neutrophil and monocyte chemotaxis toward C5a and formylated peptides (22–24, 39)	37	54	Nonchronic
TetR/AcrR family transcriptional regulator (WP_000540837.1)	*farR*	Cellular environmental dynamics monetization (40)	7	19	Nonchronic
Multidrug export protein (WP_000107169.1)	*emrB*	Part of the tripartite efflux system EmrAB-TolC, conferring antibiotic resistance^*h*^	7	19	Nonchronic
Restriction endonuclease subunit S (WP_000323907.1)	*hsdS1*	As part of a complex, restriction and modification to provide protection against foreign invaders^*i*^	5	15	Nonchronic
Deferrochelatase/peroxidase EfeB (WP_041512012.1)	*fepB*	Involved in the recovery of exogenous heme iron and shows peroxidase activity on guaiacol and catechol *in vitro*^*j*^	6	16	Nonchronic
Type 1 restriction enzyme R protein (WP_000577171.1)	*rplL*	Endonucleolytic cleavage of DNA^*k*^	16	29	Nonchronic
Inner membrane transport permease YbhR (WP_000696058.1)		Part of an ABC transporter complex, possibly involved in cefoperazone efflux and cross-membrane substrate translocation^*l*^	21	35	Nonchronic
Tetracycline resistance protein, class B (WP_000592652.1)	*norA*	Metal-tetracycline/H^+^ antiporter by energy-dependent tetracycline efflux^*m*^	7	16	Nonchronic
Zinc-type alcohol dehydrogenase-like protein (WP_000644427.1)		Oxidoreductase^*n*^	7	16	Nonchronic
DNA translocase FtsK (WP_214518494.1)		Essential coordinator during chromosome segregation and cell division (41)	7	16	Nonchronic

a Proteins with annotation as a hypothetical protein were removed (for the full list, see Table S1 in the supplemental material). “% chronic present” refers to percentage of isolates from chronic carriers that had the specific gene product, and “% nonchronic present” refers to the percentage of isolates from nonchronic carriers with the specific gene product. HTH, helix-turn-helix.

b See UniProt accession number P39647 (https://www.uniprot.org/uniprot/P39647).

c See UniProt accession number W8TR84 (https://www.uniprot.org/uniprot/W8TR84).

d See InterPro accession number IPR017782 (https://www.ebi.ac.uk/interpro/entry/InterPro/IPR017782/).

e See UniProt accession number O34362 (https://www.uniprot.org/uniprot/O34362).

f See https://aureowiki.med.uni-greifswald.de/SA_RS14160.

g See UniProt accession number O34572 (https://www.uniprot.org/uniprot/O34572).

h See UniProt accession number P0AEJ1 (https://www.uniprot.org/uniprot/P0AEJ1).

i See InterPro accession number IPR004546 (https://www.ebi.ac.uk/interpro/entry/InterPro/IPR004546/).

j See UniProt accession number Q8XAS4 (https://www.uniprot.org/uniprot/Q8XAS4).

k See UniProt accession number P08956 (https://www.uniprot.org/uniprot/P08956).

l See UniProt accession number P0AFQ1 (https://www.uniprot.org/uniprot/P0AFQ1).

m See UniProt accession number P02980 (https://www.uniprot.org/uniprot/P02980).

n See UniProt accession number Q5HM44 (https://www.uniprot.org/uniprot/Q5HM44).

Of previously described colonization-associated genes, we found only *chp* to be statistically significantly associated with nonchronic carriage (Table S2). Neither Panton-Valentine leukocidin (PVL), the arginine catabolic mobile element (ACME), *clfB*, *isdA*, *atlA*, *eap*, *tagO*, *sceD*, *oatA*, *sak*, *chp*, nor *spa* was associated with the duration of MRSA carriage.

### Machine learning predictions.

**FIG 4:**
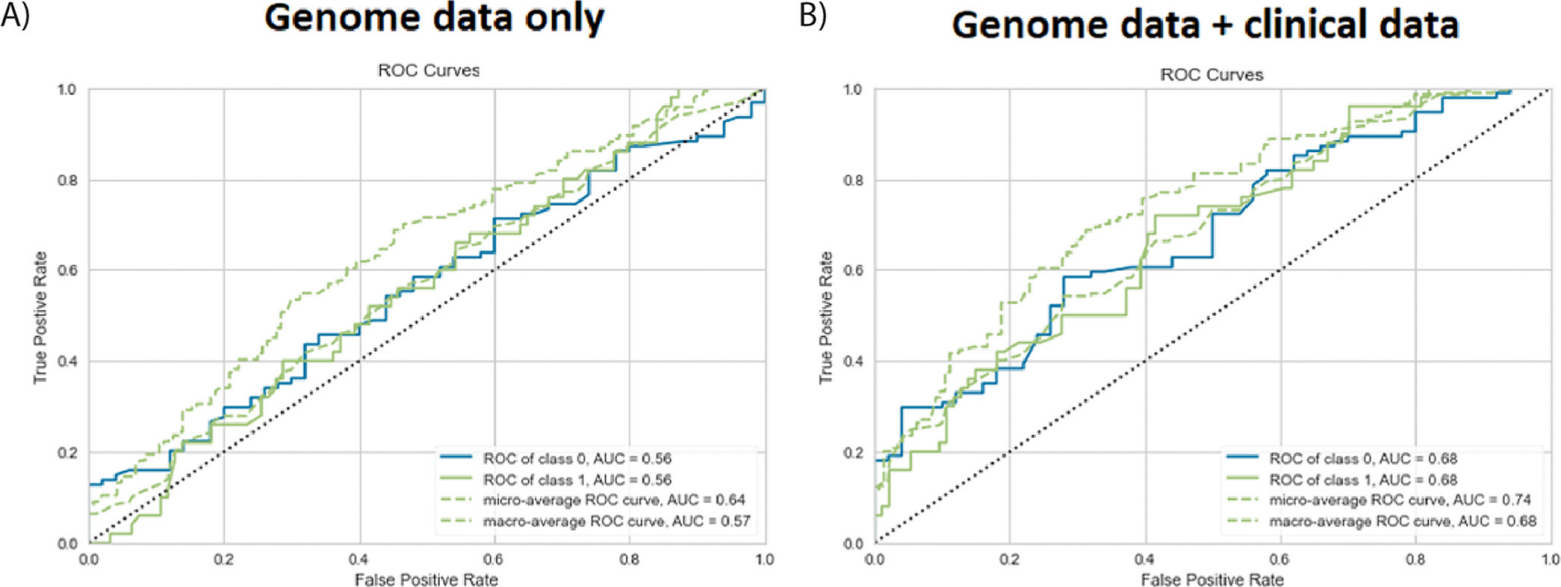
Prediction of chronic carriage using the machine learning model. (A) By incorporating genome data only, we were able to predict chronic strains with 56% accuracy. (B) The addition of clinical data to genome data increased the ability to predict chronic strains to 68% accuracy. ROC, receiver operating characteristic.

Using machine learning, an xgboost model was constructed based on bacterial genome data only. We found an ~56% prediction accuracy (Fig. 4A). When combining bacterial genome data with clinical metadata, our chronic-carriage prediction increased to 68% (area under the curve [AUC] = 0.71; precision = 0.61; recall = 0.5) (Fig. 4B).

Searching the Comprehensive Antibiotic Resistance Database (CARD) for resistance genes, we focused on resistance to mupirocin and chlorhexidine, as this would have an impact on decolonization success. Two isolates had the *mupA* gene conferring resistance to mupirocin; neither of these was obtained from patients with chronic carriage. Two other isolates had the *qacA* gene (chlorhexidine resistance), with one coming from a patient with chronic carriage.

## DISCUSSION

Our diverse bacterial isolate collection from 477 MRSA carriers (Fig. 1) enabled us to address aspects that influence MRSA decolonization. In terms of clinical data, our multivariate analysis of the clinical factors found that the three oldest age groups (ages of >13 years) were statistically associated with nonchronic carriage. At the MRSA Knowledge Center, children are typically monitored with yearly control swabs after two unsuccessful standard decolonization treatments. As we more often treat adult MRSA carriers with clindamycin and rarely offer this treatment to children, we thought that this treatment difference could influence the chronic-carrier state. The addition of clindamycin to the decolonization treatment was, however, infrequent, especially during the first treatment, and was found not to be associated with nonchronic carriage in our multivariate analysis. Two other retrospective, nonrandomized studies (10, 12) found no improvement in decolonization when antibiotics were added, but the choice of study design reveals a current gap in high-impact literature and a need for caution in the interpretation of results. A few studies have indicated promising results when systemic antibiotics are added to the decolonization treatment (13–15). Jörgensen et al. (15) found that when systemic antibiotics were added to the decolonization treatment in children between 0 and 18 years of age, 35/54 (65%) of the children were successfully decolonized, compared with 15/42 (36%) in the group with no added antibiotics. Furthermore, Jörgensen et al. found that without any decolonization treatment, 38% of carriers were spontaneously decolonized 1 year after the initial detection, and 72% were MRSA free after 2 years. These numbers, despite a lack of treatment, are considerably close to the decolonization rates in the current study, even though all carriers in our study underwent treatment, which highlights the urgent need for careful consideration regarding the effect and possible consequences of antibiotic use in asymptomatic carriers (16–18). Therefore, we are currently performing a randomized, placebo-controlled, double-blind trial in adults, registered at ClinicalTrials.gov (www.clinicaltrials.gov) under EudraCT identifier 2019-002631-29, to determine the effect of clindamycin on MRSA throat decolonization (19).

Other reasons for the seemingly higher number of chronic carriers among children (Fig. 2) could be a result of biological factors or an increased number of household members, as prolonged carriage was previously found to be associated with other household members carrying MRSA (15) but was statistically insignificant in our study. It may be more difficult to decolonize members of households with children, as the cleaning-and-washing regimen is time-consuming and demanding, possibly resulting in compromised compliance.

Surprisingly, the multivariate analysis failed to confirm that nose or throat positivity is related to whether carriage becomes chronic. Our experience in the MRSA Knowledge Center is that long-term or chronic carriers are often solely throat positive after multiple treatment attempts, and previously, it was shown that the throat is more difficult to clear (10).

As 19% of all included carriers remained MRSA positive at the end of the study period, we ideally would have wanted to know the carriers’ MSSA colonization status before acquiring MRSA, to unravel if there has been a mere replacement of MSSA with MRSA. As it was previously described that 20 to 30% of the population are persistent carriers of S. aureus (1), we wonder if these remaining MRSA-positive carriers in our study were among those 20 to 30% of persistent S. aureus carriers initially and therefore would be very difficult to treat. This question remains unanswered.

By plotting strains from chronic and nonchronic carriers against each other using cgMLST (see Fig. S1 in the supplemental material), chronic strains are scattered across the phylogenetic tree among nonchronic strains, indicating that the clonal background of a strain is not related to chronic or nonchronic carriage. In a similar grouping of isolates in an MCA plot (Fig. 3), based on genetic resemblance, we found 16%, 34%, and 45% chronic carriers in the three groups, respectively, indicating that the bacterial genome could play a role in decolonization success. Pangenome-wide association analysis found a total of 278 genetic features (for the top 20, see Table 2; for the full list, see Table S1) statistically associated with chronic or nonchronic carriage, including proteins involved in bacteriocin immunity (GO:0030153) and toxin production (GO:0009404). Among other statistically significant genes associated with chronic or nonchronic carriage, most are, unfortunately, currently scarcely described in the literature, and 143 out of the 278 genes found encoded unannotated hypothetical proteins. Comparison of data between studies is complicated by differences in the nomenclature used and inadequate knowledge about exact gene functions regarding MRSA carriage. The DNA invertase Hin was found to be the gene product most highly associated with chronic carriage, followed by a thiolase-like protein (Table 1). While the DNA invertase Hin is known to initiate DNA cleavage and recombination (20), and the thiolase-like protein is involved in the mevalonate pathway and isoprenoid biosynthesis regulation (21), the lack of a deeper understanding of their role, as well as the roles of most of the other gene products found, in MRSA colonization indicates the need for further research on this subject. We found *chp* to be the gene most highly associated with nonchronic carriage. *chp* was previously found to provide S. aureus the ability to escape the innate immune system and contribute to colonization and the early stage of infection (22–24). At first glance, our findings may seem contrary to previous findings; however, during conditional studies, Rooijakkers et al. found that when studying the transcription of *chp* in laboratory and clinical strains, an immediate exponential growth phase with high *chp* levels was quickly reached during the initial phase, followed by a quick drop once a steady-state level was reached (24). Of our isolates from chronic carriers, 61/165 (37%) had *chp*, whereas 170/312 (54%) of isolates from nonchronic carriers had *chp*, indicating that *chp* is not confined to chronic carriage but could be involved in the initial colonization phase. Apart from *chp*, surprisingly, we were unable to detect an association between other specific previously described colonization factors (Table S2) and MRSA carriage.

To further elucidate the association of the bacterial genome, we built machine learning models, with and without clinical metadata, to compare predictive outcomes, a method that, to our knowledge, has never been performed previously for the prediction of MRSA carriage. The models showed that with bacterial genomic data, we were able to predict chronic strains with 56% accuracy (Fig. 4A). By adding clinical metadata, the prediction accuracy increased to 68% (Fig. 4B), proving the significance of clinical factors. With these results, we are currently unable to use this model in practice, as it cannot provide sufficiently reliable data, useful in terms of predictions of whether a person will become a chronic carrier, and a prediction accuracy higher than 68% would be desirable. We postulate that the remaining 32% lack of predictability is caused by the lack of information on factors such as host biological factors and the influence of the different microbiomes of the nose, throat, and gut. By continuously increasing our knowledge of the above-mentioned factors as well as our understanding of significant gene products associated with chronic carriage, we may, in time, increase the ability to improve the input of data into the model and, thereby, expectedly increase the predictability output.

As mupirocin and chlorhexidine are part of the decolonization treatment, resistance to these agents would affect the decolonization rates and carrier state. However, resistance to mupirocin and chlorhexidine was very rare among carriers in this study and therefore did not affect the results of the study.

The strengths of the study include a large number of included carriers with reliable data based on the actual number of MRSA patients within our geographic area for two whole consecutive years and a long follow-up period. The analysis relies solely on carriers aspiring decolonization, leaving out all of those who were “lost to follow-up.” A weakness of the study, however, is that the decolonization rate may be inaccurate in a smaller number of the carriers, as some household members were treated before a possible carriage state was revealed since all household members are often treated simultaneously once a single MRSA carrier in a household is confirmed. Household members could have been MRSA positive and decolonized without knowing it, resulting in a falsely lower decolonization rate at the first treatment and a lower rate of nonchronic carriers. We had sparse information on comorbidities, and medical journal registration of, e.g., chronic skin disease could have been missed; however, all patients are questioned about skin diseases before commencing treatment.

### Conclusion.

Our study concludes that both the bacterial stain and the carrier, hereunder clinical and individual host biological factors, are responsible for the success or lack thereof of MRSA decolonization. Patient age and the presence of *chp* and the gene encoding the DNA invertase Hin were among the top features predictive of the duration of carriage.

We recommend that a universal clarifying term as “chronic” carriage be identified for easier comparison among data from future studies on the subject, e.g., defined as carriers who are still MRSA positive after two or more treatments, as used in this study.

## MATERIALS AND METHODS

### Ethics.

There was no direct contact with patients during this study, and no approval from ethics committees or consent from participants is required for registry-based studies in Denmark. All data were presented anonymously. Approval by the Danish Patient Safety Authority was obtained on 10 December 2019, reference EMGW, case 31-1521-65, prior to investigations, and approval by the Danish Data Protection Agency was obtained on 4 December 2019, case P-2019-784.

### Clinical data.

This study was conducted as a case-control study. The study population included all new MRSA carriers (any first-time MRSA-positive swabs from the nose, throat, and/or perineum) within the Hvidovre Hospital catchment area, handled by the laboratory in 2017 and 2018, with the intent to decolonize carriers. We defined carriers who were MRSA free after the first or second decolonization treatment as nonchronic carriers, while carriers who were still MRSA positive after two or more treatments were defined as chronic carriers. All chronic carriers from our study population were selected as cases, whereas nonchronic carriers were selected as controls.

We monitored each carrier from the first positive MRSA swab until 1 March 2020. Clinical data included sampling date, anatomical sampling site, subsequent MRSA infections, age, sex, household composition, number of decolonization treatments, and use of systemic antibiotics during MRSA decolonization. The data had been registered by doctors and infection control nurses from the MRSA Knowledge Center, Hvidovre Hospital.

### Carriers.

Each cultured isolate was confirmed to be MRSA positive by in-house multiplex real-time PCR as previously described (25). If the first sample was obtained from an infection site, the infection was initially treated. Postinfection control swabs were taken from the nose, throat, and perineum by the patients’ general practitioners (GPs) to test for MRSA carriage. Carriers with chronic wounds, active skin disease, daily livestock contact, or MRSA-positive household members below the age of 2 years were generally not offered decolonization treatment, as recommended by the DHA. Carriers who were noncompliant or did not have timely control swabs taken could not be categorized as chronic or nonchronic carriers and were excluded from this study (Fig. 1).

### Age groups.

The patients were divided into four age groups, children (2 to 12 years old), teenagers and young adults (13 to 22 years old), adults (23 to 45 years old), and older adults (above 46 years of age), to investigate the influence of age and chronic carriage.

### Decolonization regimen.

The standard decolonization treatment consisted of 2% mupirocin nasal ointment, three times daily in both nostrils; full body and hair wash using 4% chlorhexidine soap once daily for 5 consecutive days; as well as thorough cleaning. The cleaning regimen includes daily changes of towels and daily cleaning of all surfaces and contact points plus vacuuming and changes of bedlinens on days 2 and 5. All household members were asked to follow the same decolonization treatment without previous MRSA screening. One month after treatment, carriers and household members were screened from nose, throat, and perineum swabs. If MRSA positive, a new treatment was offered, this time including only MRSA-positive carriers within the household. If MRSA negative at 1 month, another screen was performed 6 months after treatment for patients to be declared MRSA free. Positive swabs at any time during the follow-up period were considered treatment failure and not a new incidence unless a new MRSA type was detected.

If the carrier was MRSA positive by throat swab, systemic antibiotics could be added to the standard treatment, usually on the second or third decolonization attempt, with clindamycin being our first-line choice.

### WGS.

We have performed WGS routinely since January 2013 on first-time MRSA isolates from all patients on a MiSeq platform (Illumina) with 150-bp paired-end reads (25). The reads from the MiSeq analysis were assembled with SPAdes (V3.11.0) (26), using default settings. Sequences were annotated using PROKKA v1.13.4 (27) and AureoWiki (28). Furthermore, Comprehensive Antibiotic Resistance Database (CARD) resistance gene identifier (RGI v5.2) software (29) was used to map antimicrobial resistance genes to genomes, including a specific search for genes conferring resistance to mupirocin and chlorhexidine. EggNOG v1.0.3 (30) was used to assign functional groups and gene ontology (GO). Multilocus sequence types (STs) were extracted from the assembled genomes using an in-house script. Core-genome multilocus sequence typing (cgMLST) was performed on all genomes using Ridom SeqSphere (V7.5.5 [April 2021]) (31) according to the published scheme for S. aureus, including 1,861 core genes. A minimum-spanning tree (MST) was constructed in Ridom SeqSphere based on cgMLST complex types (CTs).

### Pangenome construction.

Sequence homology was used to cluster genes into gene families using panX v1.60 (32) with default parameters to construct the pangenome. A pangenome can be divided into a core genome (genes shared among all strains and likely encoding functions related to basic cellular biology) and an accessory or dispensable genome (genes shared among some but not all strains and likely encoding specific advantages) (33). Gene family identifiers were assigned for each gene and used to identify the shared gene families across genomes. The presence or absence of a given gene family in a particular isolate was used to calculate statistics and train machine learning models as described below to analyze the pangenome for gene families and GO terms that were statistically associated with chronic carriage. We searched specifically for the presence of Panton-Valentine leukocidin (PVL), which was previously found to be associated with higher decolonization rates (10), as well as previously described colonization markers associated with S. aureus, *clfB*, *isdA*, *atlA*, *eap*, *tagO*, *sceD*, *oatA*, *sak*, *chp*, and *spa*, as well as the arginine catabolic mobile element (ACME) (2, 3, 34). A multiple-correspondence analysis (MCA) implemented in python was performed on the binary form of the pangenome to analyze clusters of strains.

### Statistical analyses.

The presence or absence of gene families within the pangenome was represented as binary vectors. Scripts in python were written to calculate the statistical association of each gene family with the representative metadata, including the patients’ status as chronic or nonchronic carriers. Both Fisher’s exact tests and chi-squared tests were used to calculate *P* values for all tests. Statistical significance was defined as those features with calculated Fisher’s exact test *P* values of 0.05 or lower. Cramer’s *V* statistic for categorical-to-categorical association was also used to evaluate associations between pangenome clusters and STs. This calculation was implemented in python and used the correction described previously by Bergsma (35).

A multivariate logistic regression analysis was performed and expressed as odds ratios (ORs) with 95% confidence intervals (CIs), to evaluate the combined contribution of each factor to chronic carriage. The logistic regression incorporated age, household size, clindamycin treatment (first or second treatment), gender, and whether patients were positive in the nose and/or throat (first positive site).

Observations with missing values were excluded from the analysis.

### Machine learning.

The binary form of the pangenome was used as an input to construct a gradient boost machine learning model where each feature was a binary vector representing the presence or absence of a gene family within a given strain’s genome. Patient metadata (household, gender, location of the positive swab, and age) were included as features for the combined machine learning models. The machine learning models were implemented in scikit-learn as a gradient boost classifier with 10× cross-validation bootstraps, with each iteration containing 30% of data held out for the test set.

### Data availability.

MRSA sequence reads have been deposited in GenBank under BioProject accession number PRJNA865897 and SRA accession numbers SRR13767899, SRR13767894, SRR19351883, and SRR19351716.
