# Supplementary material for: Estimated Roles of the Carrier and the Bacterial Strain When Methicillin-Resistant Staphylococcus aureus Decolonization Fails: a Case-Control Study
Source: Microbiol Spectr. 2022 Aug 24;10(5):e01296-22. doi: 10.1128/spectrum.01296-22 (PMC9602359; doi:10.1128/spectrum.01296-22)

SUPPLEMENTARY

Supplementary table 1

Full list of significant genetic features ($p < 0.05$) in alphabetical order – unannotated hypothetical proteins were removed

Gene Product	Carriage Association	NCBI Protein ID
Adenosyl-chloride synthase	Chronic	WP_000528922.1
Aldehyde-alcohol dehydrogenase	Chronic	WP_000955781.1
Alkylmercury lyase	Chronic	WP_001011857.1
Aminoglycoside 3'-phosphotransferase	Chronic	WP_001096887.1
Bifunctional ligase/repressor BirA	Chronic	WP_001079936.1
Bleomycin resistance protein	Non-chronic	WP_001242578.1
Chemotaxis inhibitory protein	Non-chronic	WP_000727649.1
DegV domain-containing protein	Non-chronic	WP_001118605.1
Dihydrofolate reductase type 1 from	Non-chronic	WP_000175735.1
DNA processing protein DprA variant 1	Non-chronic	WP_000230031.1
DNA processing protein DprA variant 2	Non-chronic	WP_000931873.1
DNA translocase FtsK	Non-chronic	WP_214518494.1
DNA-invertase hin	Chronic	WP_000690626.1
dTDP-fucosamine acetyltransferase	Chronic	WP_057098418.1
Enterotoxin type A variant 1	Chronic	WP_000750406.1
Enterotoxin type A variant 2	Non-chronic	WP_001235656.1
Enterotoxin type B	Non-chronic	WP_000764684.1
Enterotoxin type D variant 1	Non-chronic	WP_000935747.1
Enterotoxin type D variant 2	Non-chronic	WP_000821658.1
Enterotoxin type G	Non-chronic	WP_000736712.1
Epidermin decarboxylase	Chronic	WP_000504439.1
ESAT-6 secretion machinery protein EssD	Chronic	WP_000159026.1
ESAT-6 secretion system extracellular protein B	Chronic	WP_000509667.1
ESAT-6 secretion system extracellular protein C	Chronic	WP_001010291.1
ESAT-6 secretion system extracellular protein D	Chronic	WP_000175723.1
Extracellular matrix protein-binding protein emp	Non-chronic	WP_000824488.1
Extracellular metalloprotease	Non-chronic	WP_000161219.1
Glutamine transport ATP-binding protein GlnQ	Chronic	WP_228587459.1
Glycyl-glycine endopeptidase ALE-1	Non-chronic	WP_000504558.1
Group II intron-encoded protein LtrA	Non-chronic	WP_052997621.1
HTH-type transcriptional regulator BetI	Chronic	WP_001124173.1
HTH-type transcriptional regulator CysL variant 1	Chronic	WP_000377738.1
HTH-type transcriptional regulator CysL variant 2	Chronic	WP_000371597.1
HTH-type transcriptional regulator YofA	Non-chronic	WP_001010294.1
Hydroxyacylglutathione hydrolase	Chronic	WP_000184398.1

Immunodominant staphylococcal antigen B variant 1	Non-chronic	WP_001077099.1
Immunodominant staphylococcal antigen B variant 2	Chronic	WP_001044560.1
Inner membrane transport permease YbhR	Non-chronic	WP_000696058.1
Insertion sequence IS5376 putative ATP-binding protein	Non-chronic	WP_001066125.1
Kanamycin nucleotidyltransferase	Non-chronic	WP_223296184.1
Lantibiotic gallidermin	Chronic	WP_000416756.1
Malolactic enzyme	Non-chronic	WP_074371004.1
Maltose O-acetyltransferase	Chronic	WP_000636149.1
Modification methylase FokI	Chronic	WP_016169267.1
Multidrug export protein EmrB	Non-chronic	WP_000107169.1
Nisin biosynthesis protein NisB	Chronic	WP_001092606.1
Nisin biosynthesis protein NisC	Chronic	WP_000566596.1
Nisin leader peptide-processing serine protease NisP	Chronic	WP_000691541.1
Plasmid recombination enzyme	Chronic	WP_000390798.1
Poly(glycerol-phosphate) alpha-glucosyltransferase	Non-chronic	WP_052997135.1
putative ABC transporter ATP-binding protein YbhF	Non-chronic	WP_000340149.1
putative ABC transporter ATP-binding protein YxlF	Chronic	WP_117201003.1
putative ABC transporter ATP-binding protein YxlF	Chronic	WP_001059585.1
putative antitoxin YezG variant 1	Chronic	WP_000142122.1
putative antitoxin YezG variant 2	Chronic	WP_049311680.1
putative ATP-dependent helicase DinG	Chronic	WP_001566849.1
putative cell wall hydrolase LytN	Chronic	WP_072528212.1
Putative HMP/thiamine import ATP-binding protein YkoD	Chronic	WP_000138663.1
Putative HMP/thiamine permease protein YkoC	Chronic	WP_001077829.1
putative isomerase YddE	Chronic	WP_000708541.1
putative lipoprotein	Non-chronic	WP_000581887.1
putative lipoprotein YehR	Chronic	WP_000758108.1
putative N-acetyltransferase YjaB	Chronic	WP_045177903.1
RecBCD enzyme subunit RecD	Chronic	WP_042747172.1
Response regulator protein GraR	Chronic	WP_000866106.1
Sensor histidine kinase GraS	Chronic	WP_000270041.1
Streptogramin A acetyltransferase	Non-chronic	WP_001216898.1
Tetracycline resistance protein, class B	Non-chronic	WP_000592652.1
Thymidylate synthase	Non-chronic	WP_000282655.1
Toxic shock syndrome toxin-1	Non-chronic	WP_001035599.1
Transcriptional regulator SlyA	Chronic	WP_158176026.1
Transposase from transposon Tn916 Variant 1	Non-chronic	WP_061838911.1
Transposase from transposon Tn916 Variant 2	Non-chronic	WP_001145728.1
Type I restriction enzyme EcoKI M protein	Non-chronic	WP_000190897.1
Type-1 restriction enzyme R protein Variant 1	Non-chronic	WP_000577171.1
Type-1 restriction enzyme R protein Variant 2	Non-chronic	WP_001010920.1
Type-2 restriction enzyme FokI	Chronic	WP_000877434.1
Tyrosine recombinase XerC variant 1	Non-chronic	WP_000706211.1
Tyrosine recombinase XerC variant 2	Non-chronic	WP_000026854.1
Tyrosine recombinase XerD variant 1	Chronic	WP_049311812.1
Tyrosine recombinase XerD variant 2	Chronic	WP_001044915.1

Undecaprenyl-diphosphatase	Chronic	WP_061491645.1
Zinc-type alcohol dehydrogenase-like protein	Non-chronic	WP_000644427.1

Supplementary Table 2

Genes associated with colonization from literature explored

**Gene**	Gene product	p-value	Carriage association
chp	Chemotaxis inhibitory protein	<0.05	Non-chronic
clfB	Clumping factor B	>0.05	-
atl	Bifunctional autolysin	>0.05	-
eap/map	65 kDa membrane protein	>0.05	-
spa	Immunoglobulin G-binding protein A	>0.05	-
isdH	Iron-regulated surface determinant protein H	>0.05	-
atl	Bifunctional autolysin	>0.05	-
sak	Staphylokinase	>0.05	-
PVL	Panton-Valentine Leukocidin	>0.05	-
ACME	Arginine catabolic mobile element	>0.05	-
isdA	Iron-regulated surface determinant protein A	NA	NA
tagO	Undecaprenyl-phosphate alpha-N-acetylglucosaminyl 1-phosphate transferase	NA	NA
sceD	Transglycosylase SceD	NA	NA
oatA	O-acetyltransferase OatA	NA	NA
**NA = found in all strains**			

Supplementary figure 1

cgMLST based minimum spanning tree of chronic (orange) and non-chronic (blue) isolates, show chronic strains scattered randomly.

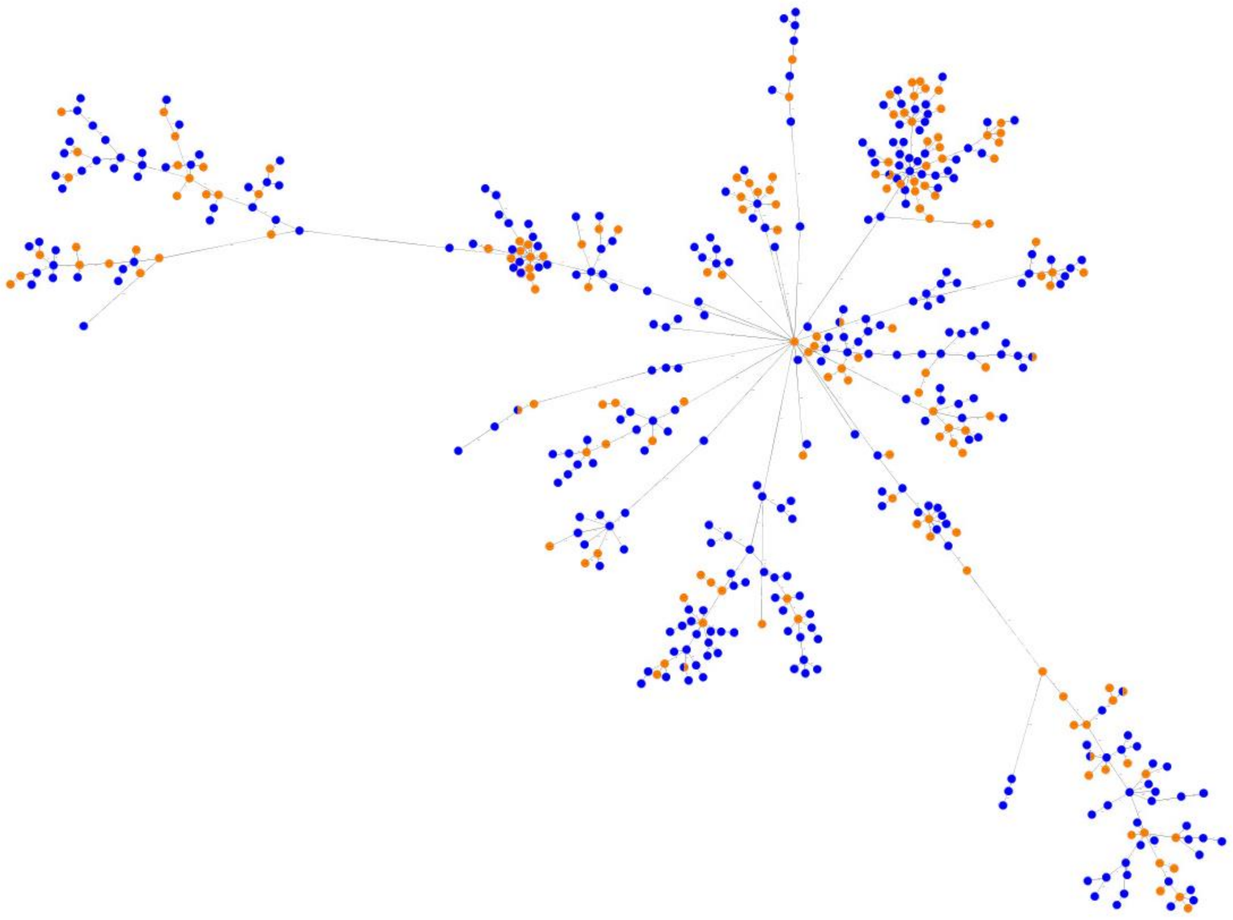

Supplement: Supplemental file 1 — Tables S1 and S2 and Fig. S1. Download spectrum.01296-22-s0001.pdf, PDF file, 0.2 MB [file spectrum.01296-22-s0001.pdf]
